# Pediatric Invasive Meningococcal Disease, Auckland, New Zealand (Aotearoa), 2004–2020

**DOI:** 10.3201/eid2904.221397

**Published:** 2023-04

**Authors:** Cameron Burton, Emma Best, Matthew Broom, Helen Heffernan, Simon Briggs, Rachel Webb

**Affiliations:** The University of Auckland, Auckland, New Zealand (C. Burton, E. Best, R. Webb);; Te Whatu Ora Counties Manukau, Auckland (C. Burton, R. Webb);; Te Whatu Ora Te Toka Tumai Auckland, Auckland (E. Best, S. Briggs, R. Webb);; Te Whatu Ora Waitematā, Auckland (M. Broom);; Institute of Environmental Science and Research, Wellington, New Zealand (H. Heffernan).

**Keywords:** Meningococcal disease, pediatric, New Zealand, *Neisseria meningitidis*, sepsis, bacteremia, meningitis/encephalitis, bacterial infections, health disparity, bacteria

## Abstract

New Zealand (Aotearoa) experienced a *Neisseria meningitidis* serogroup B epidemic during 1991–2006, and incidence remains twice that of other high-income countries. We reviewed clinical, laboratory, and immunization data for children <15 years of age with laboratory-confirmed invasive meningococcal disease in Auckland, New Zealand, during January 1, 2004–December 31, 2020. Of 319 cases in 318 children, 4.1% died, and 23.6% with follow-up data experienced sequelae. Children of Māori and Pacific ethnicity and those living in the most deprived areas were overrepresented. Eighty-one percent were positive for *N. meningitidis* serogroup B, 8.6% for serogroup W, 6.3% for serogroup C, and 3.7% for serogroup Y. Seventy-nine percent had bacteremia, and 63.9% had meningitis. In New Zealand, Māori and Pacific children are disproportionately affected by this preventable disease. *N. meningitidis* serogroup B vaccine should be included in the New Zealand National Immunization Schedule to address this persistent health inequity.

## Abstract 2

Māori and Pacific children are disproportionately affected by this preventable disease.

Invasive meningococcal disease (IMD) is a bacterial infection with typically rapid onset. In children, infection is associated with high (7%–9%) case-fatality rates (CFRs) and serious long-term sequelae (*1*,*2*). Infants and young children have the highest incidence of disease; a second peak occurs during adolescence (*3*). IMD inequitably affects Indigenous populations and persons living in areas of deprivation (*3*,*4*). 

The bacterium *Neisseria meningitidis* is categorized into serogroups based on its polysaccharide capsule; 6 serogroups (A, B, C, W, X, and Y) are responsible for nearly all IMD cases worldwide (*5*). The major clinical manifestations of IMD are meningitis and sepsis. Early recognition is critical because sepsis can rapidly progress to multiorgan dysfunction and death (*6*). A leading cause of admission to pediatric intensive care units (ICUs) throughout Australasia (*7*), IMD can lead to disabling, long-term sequelae for approximately one third of surviving children, including hearing loss, neurodevelopmental impairment, limb or digit loss, and scarring (*2*,*8*,*9*). Those sequelae heavily affect healthcare resources and the quality of life of affected children and their families (*2*,*9*).

## Epidemiology of IMD Globally and in New Zealand

The global incidence of IMD has declined over the past 20 years, partly because of the availability of safe, effective vaccines for all major disease-causing serogroups and successful vaccination programs (*5*). Overall incidence of IMD in most high-income countries is well under 1.5 per 100,000 per year (*5*). In contrast, New Zealand (Aotearoa) reports the highest rate of *N. meningitidis* serogroup B (MenB) disease in the world (*3*,*5*,*10*). During 1991–2006, New Zealand experienced a prolonged MenB epidemic caused by the B:P1.7–2,4 strain (*11*). The epidemic peaked in 2001, with an incidence of 17.4 cases/100,000 persons in the overall population and 212 cases/100,000 infants (*11*). In response, MeNZB, a strain-specific outer membrane vesicle (OMV) vaccine, was developed and delivered nationally in 3 doses to persons <20 years of age during 2004–2006 and in 4 doses to infants during 2006–2008 (*11*). Overall vaccination coverage was 80%, and coverage was higher among Pacific peoples compared with those of other ethnicities. The vaccine effectiveness of MeNZB against the epidemic strain was estimated at 68%–77% and was associated with the waning of the epidemic (*4*,*11*). 

Since that time, regional outbreaks of *N. meningitidis* serogroup C (MenC) and serogroup W (MenW) disease have been associated with high CFRs, prompting emergency targeted vaccination programs in 2011 and 2018 (*12*,*13*). However, since 2014, the incidence of IMD in NZ has been increasing, up to an overall rate of 2.8 cases/100,000 persons in 2019 (*3*). Almost half of cases in 2019 occurred in children <15 years of age, and the highest rates in infants <1 year of age (51.5/100,000 infants). As observed internationally, an increasing proportion of IMD caused by MenW has occurred in New Zealand, accounting for 30% of the country’s cases in 2019 (*3*,*5*). Auckland, New Zealand’s largest city, has a pediatric (<15 years of age) population of ≈320,000, which makes up 34% of the total New Zealand pediatric population (*14*). Ethnic groups in Auckland include Māori (18%), Pacific peoples (19%), and those of Asian (25%) and European (34%) heritage. 

## Meningococcal Vaccines

A 4-component MenB vaccine, 4CMenB (Bexsero; GlaxoSmithKline), was developed using 3 subcapsular antigens and the NZ MeNZB OMV vaccine (*15*). Vaccine effectiveness data from Australia, Canada, Italy, and the United Kingdom show reductions in MenB of 71%–100% in eligible cohorts 2–5 years after 4CMenB was introduced (*15*). Although there is no evidence that 4CMenB reduces *N. meningitidis* carriage (*16*), OMV meningococcal vaccines appear to provide some protection against IMD caused by non-MenB serogroups, as well as against *N. gonorrhoeae* (*17*,*18*). Although 4CMenB and MenACWY vaccines are funded in New Zealand for a small number of persons with high-risk medical conditions and, recently, for adolescents in certain collective residences, no meningococcal vaccines are universally funded in the National Immunization Schedule. We aimed to describe the experience of pediatric IMD in Auckland during 2004–2020—including demographic factors; clinical, microbiological, and laboratory features; treatment; and outcomes—to demonstrate the impact of IMD on children in New Zealand and to highlight the need for funding of meningococcal vaccines.

## Methods

### Study Design and Collection of Data

We conducted a retrospective, observational study in Auckland. Eligible cases were those in children <15 years of age who contracted IMD while residing within the Auckland region during January 1, 2004–December 31, 2020. We included cases where *N. meningitidis* was identified by culture or PCR from a normally sterile site (i.e., blood, cerebrospinal fluid [CSF], synovial fluid). 

All persons who test positive for *N. meningitidis* in New Zealand are actively notified as part of public health surveillance; isolates and DNA extracted from sterile site specimens are forwarded to the Meningococcal Reference Laboratory at the Institute of Environmental Science and Research (*3*). The institute provided all cases confirmed by *N. meningitidis* culture or PCR. We collected data by using National Health Index numbers (a unique identifier for medical care for all persons residing in New Zealand) (*19*) from clinical and laboratory records and the National Immunization Register (an electronic record of vaccination events for New Zealand children) (*20*).

### Case Definitions and Variables

We categorized clinical manifestations according to the presence of bacteremia, meningitis, and septic arthritis. We defined bacteremia as a positive *N. meningitidis* culture or PCR from blood. We defined meningitis as a positive *N. meningitidis* culture or PCR from CSF or an alternative sterile site positive for *N. meningitidis* with a CSF leukocytosis or with clinical signs of meningitis if CSF was not obtained. We defined septic arthritis as a positive *N. meningitidis* culture or PCR from synovial fluid or an alternative sterile site positive for *N. meningitidis* with clinical signs of septic arthritis. We defined sepsis by Pediatric Sepsis Consensus Congress criteria (*21*). We calculated CFR as the number of children who died divided by the total number of cases. For survivors, we classified outcomes as cure, cure with sequelae, and unknown. We used a composite outcome of death and cure with sequelae in our outcome analysis. 

We obtained population denominators from Statistics New Zealand (*22*) and recorded prioritized ethnicity using New Zealand ethnicity data protocols (*23*). We measured socioeconomic deprivation using the New Zealand Index of Deprivation (NZDep) quintiles for 2013 and 2018 (*24*). NZDep stratifies small geographic areas into equal-sized groups based on multiple measures of socioeconomic deprivation. We identified serogroup by serological means or by PCR. DNA sequence analysis of the porA gene determined the subtype. We defined the epidemic strain as MenB with the P1.7–2,4 subtype and defined vaccine subtype IMD as any serogroup with the P1.7–2,4 subtype. We determined MICs by using Etest (bioMérieux). We categorized isolates with penicillin MICs of >0.06 mg/L as having reduced penicillin susceptibility and interpreted ceftriaxone, ciprofloxacin, and rifampin MICs according to standardized breakpoints (*25*). We defined MeNZB vaccination status as fully vaccinated (received all approved doses for age), partially vaccinated (received less than approved doses for age), unvaccinated (received no doses), or ineligible (born outside of the MeNZB program period). We obtained approval for the study from the Health and Disability Ethics Committees (18/NTA/86/AM02).

### Statistical Methods

We performed calculations using R (The R Foundation for Statistical Computing, https://www.r-project.org) and OpenEpi (Open Source Epidemiologic Statistics for Public Health, https://www.openepi.com). We included only cases with available data in the analysis of each variable. We employed a 2-tailed test to determine p values, using a significance level of 0.05, and used a Poisson model to investigate temporal trends in IMD and the epidemic strain. We used univariate logistic regression to investigate factors associated with an increased risk of death or sequelae and χ^2^ test to compare rates and calculate 95% CIs. We compared MeNZB vaccination status with timing of IMD illness by using analysis of variance and independent samples t-tests.

## Results

### Case Numbers

**Figure 1:**
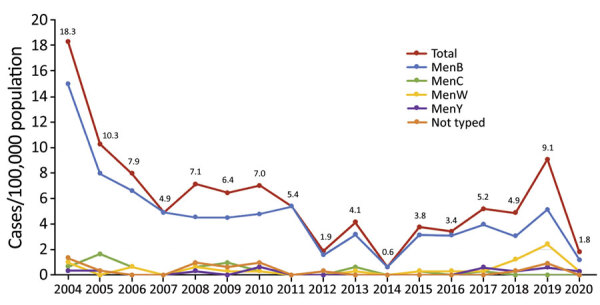
Timeline of 319 cases of confirmed invasive meningococcal disease in children <15 year of age, by serogroup, reported by year, Auckland, New Zealand, 2004–2020. Numbers along data line indicate exact rates for all cases by year. Men, *Neisseria meningitidis* serogroup.

We reviewed data from 331 cases, excluding 12 cases (6 in nonresidents, 5 that were noninvasive disease, and 1 that lacked sufficient data). The remaining 319 cases of laboratory-confirmed IMD occurred in 318 children. One child had 2 unrelated episodes of IMD that occurred in 2006 and 2017. There were no documented relapses after treatment in the cohort. The average annual incidence of IMD across the study period was 5.9/100,000 population. Incidence rates declined from the tail end of the epidemic in 2004 to a nadir in 2014, then increased to a second peak in 2019 (Figure 1). Overall, we found a trend toward reduced incidence over the study period (Poisson coefficient −0.07 [95% CI −0.14 to −0.01; p<0.01]; rate ratio 0.92 [95% CI 0.90–0.95]). Cases were more common in winter (135/319, 42.3%), followed by spring (87/319, 27.3%), autumn (52/319, 16.3%), and summer (45/319, 14.1%) (p<0.0001).

### Demographic Factors

Median age at time of diagnosis was 18 months (interquartile range [IQR] 7–60 months). The highest average incidence rates were among infants <1 year of age (31.5/100,000 population/year), followed by those 1–4 years of age (8.2/100,000 population/year), 5–9 years of age (2.6/100,000 population/year), and 10–14 years of age (2.0/100,000 population/year) (Table 1). Average incidence rates by ethnic group were highest in Pacific peoples (13.4/100,000 population/year), followed by Māori (11.6/100,000 population/year) and those who were neither Māori or Pacific (1.9/100,000 population/year) (Table 1). Based on census data and compared with non-Māori and non-Pacific groups, the unadjusted relative risk of IMD was 5.9 (95% CI 4.4–8.1) for Māori (p<0.0001) and 6.9 (95% CI 5.1–9.3) for Pacific peoples (p<0.0001). Most children (189/317, 59.6%) lived in NZDep quintile 5 (most deprived 20%) areas. The unadjusted relative risk of IMD for children living in NZDep quintile 5 areas compared with quintile 1 areas was 17.2 (95% CI 9.7–33.2; p<0.0001).

**Table 1:** Demographic and clinical factors of 319 confirmed cases of invasive meningococcal disease in children <15 years of age, Auckland, New Zealand, 2004–2020*

Variable	Total no. (missing data)	No. (%) cases	Average incidence, cases/100,000 population/y	
Age, y	319 (0)		
<1 y		118 (37.0)	31.5
1–4		120 (37.6)	8.2
5–9		46 (14.4)	2.6
10–14		35 (11.0)	2.0
Sex	319 (0)		
F		125 (39.2)	4.8
M		194 (60.8)	7.0
Ethnicity, prioritized	319 (0)		
Māori		114 (35.7)	11.6
Pacific		140 (43.9)	13.4
Non-Māori, non-Pacific		65 (20.4)	1.9
New Zealand Index of Deprivation Quintile†	317‡		
1		11 (3.5)	NA
2		21 (6.6)	NA
3		42 (13.2)	NA
4		54 (17.0)	NA
5		189 (59.6)	NA
Clinical manifestations	319 (0)		
Bacteremia only		108 (33.9)	NA
Meningitis only		63 (19.7)	NA
Meningitis with bacteremia		138 (42.3)	NA
Septic arthritis only		5 (1.6)	NA
Septic arthritis with bacteremia		2 (0.6)	NA
Meningitis and septic arthritis with bacteremia		3 (0.9)	NA
Vital signs on first presentation			
Temperature >38·5°C or <36°C	314 (5)	150 (47.7)	NA
Systolic hypotension for age	218 (101)	84 (38.5)	NA
Impaired level of consciousness	291 (28)	99 (34.0)	NA
Clinical signs at first presentation			
Rash in cases with bacteremia	248 (3)	213 (85.9)	NA
Includes purpura	213 (3)	108 (50.7)	NA
Includes petechiae without purpura	213 (3)	86 (40.4)	NA
Blanching only	213 (3)	19 (8.9)	NA
Meningism in cases with meningitis	184 (20)	111 (60.3)	NA
Bulging fontanelle in infants with meningitis	43 (39)	19 (44.2)	NA
Arthritis during admission	314 (5)	19 (6.1)	NA
Arthralgia during admission	314 (5)	23 (7.3)	NA
*NA, not applicable. †Each NZDep quintile contains ≈20% of the population. 1 = least deprived; 5 = most deprived. ‡Two overseas cases were excluded.				

### Microbiology and Laboratory Features

**Figure 2:**
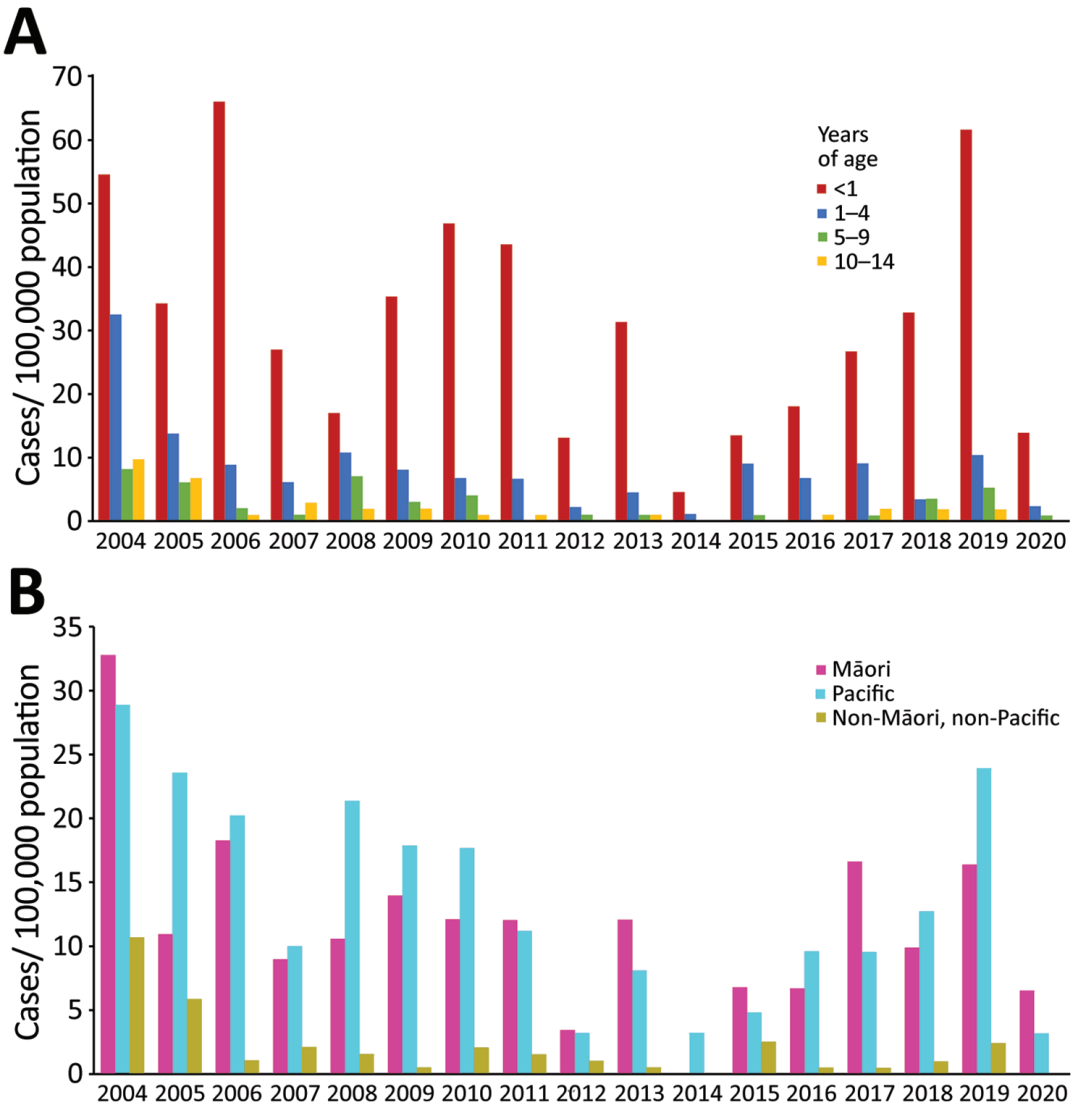
Timeline of 319 cases of confirmed invasive meningococcal disease in children <15 years of age by age group (A) and prioritized ethnicity (B), reported by year, Auckland, New Zealand, 2004–2020.

Of the 319 cases, we confirmed a microbiological diagnosis by both culture and PCR for 81 (25.4%), on culture alone for 114 (35.7%), and on PCR alone for 124 (38.9%). We compared *N. meningitidis* culture and PCR from blood and from CSF (Appendix Table 1). Blood culture was negative for 56 (78.9%) of 71 cases in children who received antibiotics before hospital admission (odds ratio 5.1 [95% CI 2.7–9.5]) compared with no prehospital antibiotics (p<0.0001). Of those 56 cases, *N. meningitidis* blood PCR was positive in all 50 cases tested. CSF analysis was performed in 138 (67.6%) of the 204 cases classified as meningitis (Appendix Table 2). CSF leukocytosis for age was present in 130 (97.7%) of 133 cases of meningitis where a CSF leukocyte count was performed. The serogroup was identified for 301 (94.4%) of the 319 cases: 245 (81.4%) were MenB, 26 (8.6%) MenW, 19 (6.3%) MenC, and 11 (3.7%) serogroup Y. Beginning in 2017, there was an increase in disease caused by MenW, which accounted for 8 (29.6%) of the 27 cases serogrouped in 2019; MenB was the remaining predominant serogroup (Figure 2). The epidemic B:P1.7–2,4 strain accounted for 135 cases (44.9%), waning over time, from 42 cases (14.0/100,000 population) in 2004 to 3 cases (0.90/100,000 population) in 2020 (Poisson coefficient −0.20 [95% CI −0.28 to −0.11]; p<0.01; rate ratio 0.82 [95% CI 0.78–0.85]). The proportion of isolates with reduced penicillin susceptibility increased during the study period. A MIC of >0.06 mg/L was identified in 28 (22.6%) of 124 isolates in 2004–2012 and 47 (66.2%) of 71 isolates in 2013–2020 (p<0.0001). Reduced penicillin susceptibility was identified in 20 (76.9%) of 26 MenW isolates compared with 55 (32.5%) of 169 non-MenW isolates (p = 0.012). All isolates were susceptible to ceftriaxone, ciprofloxacin, and rifampin.

### Clinical Features

The median duration of illness before care was sought was 1 day (IQR 1–3 days). Bacteremia was present in 251 cases (78.7%), meningitis in 204 (63.9%), and septic arthritis in 10 (3.1%) (Table 1). Concomitant bacteremia and meningitis occurred in 141 (44.2%) cases. No cases of chronic meningococcemia were recorded. Sepsis occurred in 172 (80.8%) of the 213 cases with complete systemic inflammatory response syndrome data. 

### Treatment

Of the 319 cases, 317 (99%) were treated in a hospital; 2 children (0.6%) died before arrival. The median duration of hospitalization was 5 nights (IQR 3–7 nights). Prehospital parenteral antibiotics were administered in 52 (16.3%) cases. In the hospital, empiric antibiotics included a third-generation cephalosporin in 294 (92.7%) cases. There were 303 children who completed a full targeted treatment course of antibiotics, which included a third-generation cephalosporin in 230 cases (75.9%), benzylpenicillin in 67 cases (22.1%), amoxicillin in 8 cases (2.6%), and another antibiotic in 4 cases (1.3%). The median duration of antibiotic treatment was 5 days (IQR 5–7 days). Dexamethasone was administered in 47 (23%) of the 204 meningitis cases. Of the 100 (31.3%) children who were admitted to an ICU (median duration of stay 1 night [IQR 1–3 nights]), 55 received >1 life-saving measure: 46 received invasive ventilation, 44 received inotropic/vasopressor support, and 7 received renal replacement therapy. Plastic surgical procedures were performed in 12 (3.8%) cases, orthopedic procedures in 12 (3.8%), and neurosurgical procedures in 6 (1.9%).

### Outcomes

Thirteen children died, resulting in a CFR of 4.1% (Table 2). The average death rate over the study period was 0.24/100,000 population/year. Of the 13 children who died (Appendix Table 3), 12 (92.3%) were Māori or Pacific peoples, 11 (84.6%) were living in NZDep quintile 5 areas, and 9 (69.2%) were infants <1 year of age. Ten deaths (76.9%) occurred in the community or within the first 48 hours of hospitalization. Of the 306 survivors, outcome data were complete for 258 cases (84.3%): cure without sequelae occurred in 197 (76.4%) and cure with sequelae 61 (23.6%). We classified outcome as unknown in 48 cases; all had meningitis with no available audiometry results (none had other sequelae identified on follow-up). Documented audiologic assessment occurred in 143 (72.6%) of 197 cases after meningitis. Of the 142 cases with audiometry results available, 32 (22.5%) had sensorineural impairment. Māori children with IMD had 2.5 (95% CI 1.1–6.4) times the odds for death or sequelae compared with non-Māori, non-Pacific peoples (p = 0.0366) (Table 3). Pacific peoples with IMD had 2.9 (95% CI 1.3–7.2) times the odds for death or sequelae compared with non-Māori, non-Pacific peoples (p = 0.0128). Results of univariate comparisons of age, sex, NZDep quintile, season, MeNZB vaccination status, sepsis criteria, serogroup, reduced penicillin susceptibility, and prehospital parenteral antibiotics were not significant.

**Table 2:** Outcomes of 319 confirmed cases of invasive meningococcal disease in children <15 years of age, Auckland, New Zealand, 2004–2020

Outcome	No. cases/total no. (%)
Died	13/319 (4.1)
Cure, complete outcome data	258/306 (84.3)
Cure, incomplete outcome data	48/306 (15.6)
Cure without sequelae	197/258 (76.4)
Cure with sequelae	61/258 (23.6)
Sequelae	
Neurodevelopmental	35/258 (13.6)
Sensorineural hearing loss	32/258 (12.4)
Skin scarring	16/258 (6.2)
Loss of limbs or digits	7/258 (2.7)
Chronic kidney disease	1/258 (0.4)
Other sequelae*	5/258 (1.9)
Neurodevelopmental sequelae	
Delayed development	20/258 (7.8)
Cerebral ischemia	13/258 (5)
Epilepsy	8/258 (3.1)
Learning, concentration, behavior, psychological	8/258 (3.1)
Other†	10/258 (3.9)

*Other: bone growth arrest 2/258 (0.8%); cardiomyopathy 1/258 (0.4%); gastrointestinal hemorrhage 1/258 (0.4%); panniculitis 1/258 (0.4%). †Other neurodevelopmental: chronic hydrocephalus 2/258 (0.8%); autism spectrum disorder 1/258 (0.4%); ataxia 1/258 (0.4%); carotid artery narrowing 1/258 (0.4%); chronic headache 1/258 (0·4%); cranial nerve palsy 1/258 (0.4%); encephalomalacia 1/258 (0.4%); hypertonia 1/258 (0.4%); syringomyelia 1/258 (0.4%).

**Table 3:** Univariate logistic regression for combined outcome of death or sequelae in 271 confirmed cases of invasive meningococcal disease in children <15 years of age, Auckland, New Zealand, 2004–2020*

Variable	No. cases (%)	OR (95% CI)	p value
Ethnicity, compared with non-Māori, non-Pacific population			
Pacific	38/118 (32.2)	2.91 (1.31–7.18)	0.0128
Māori	28/96 (29.2)	2.52 (1.10–6.35)	0.0366
Reduced penicillin susceptibility	12/64 (18.8)	0.548 (0.25–1.14)	0.117
NZDep quintile†	271	1.21 (0.95–1.58)	0.142
Age, mo†	271	0.996 (0.99–1.00)	0.157
Serogroup, compared with MenB			
MenC	7/18 (38.9)	1.80 (0.64–4.82)	0.247
MenW	6/25 (24.0)	0.89 (0.31–2.24)	0.822
MenY	3/8 (37.5)	1.70 (0.34–7.16)	0.478
Male sex, compared with female	50/168 (29.8)	1.39 (0.80–2.48)	0.248
Season, compared with autumn			
Spring	23/76 (30.3)	1.47 (0.64–3.60)	0.374
Summer	11/35 (31.4)	1.56 (0.57–4.31)	0.386
Winter	30/116 (25.9)	1.19 (0.54–2.79)	0.683
MeNZB vaccination, compared with fully vaccinated			
Unvaccinated	43/166 (25.9)	0.76 (0.39–1.51)	0.425
Partially vaccinated	12/41 (29.3)	0.90 (0.37–2.17)	0.817
Prehospital parenteral antibiotic treatment	10/44 (22.7)	0.79 (0.35–1.64)	0.537
Sepsis criteria	39/145 (26.9)	1.14 (0.51–2.77)	0.751

*Men, *Neisseria meningitidis* serogroup; OR, odds ratio; NZDep, New Zealand Index of Deprivation †Continuous variable; OR represents increase in odds for each unit increase in variable.

### MeNZB Vaccination

Of the 163 children with complete vaccination records who were eligible for MeNZB, 114 (69.9%) had received >1 dose and 64 (39.3%) were fully vaccinated at time of hospital admission. For the 97 eligible children with vaccine subtype IMD, 55 (56.7%) had received >1 dose and 31 (32%) were fully vaccinated at time of hospital admission. The mean number of days between the date of last MeNZB vaccine and IMD onset increased with the number of doses received (p<0.00027) (Appendix Table 4).

## Discussion

### Key Findings

Despite a reduction in the number of cases of IMD since the MenB epidemic, the incidence of IMD in New Zealand remains double that of other high-income countries (*3*,*5*). Although MenB remains the most common serogroup in children, the epidemic B:P1.7–2,4 strain no longer dominates in the Auckland region. Rates of pediatric IMD increased in Auckland and nationally in 2014–2019, partly because of an observed global increase in MenW (*3*,*5*). Mirroring international trends in invasive bacterial disease during the COVID-19 pandemic (*26*–*28*), there was a sharp decrease in cases of pediatric IMD in NZ in 2020 after national COVID-19 control measures began, and that decrease continued through 2021 (*29*). Future patterns of pediatric IMD remain uncertain; however, there is a risk of resurgent disease exacerbated by rising poverty and socioeconomic inequity (*30*).

Our findings highlight the severity of IMD. One third of the cases we studied included admission to an ICU, comparable with data for international cohorts (*8*,*31*). Over half of those cases required invasive ventilation or inotropic/vasopressor support. The CFR in our cohort was 4.1%, which compares to rates for other high-income settings of 2%–12% (*1*). Sequelae occurred in 23.6% of survivors. Because outcome was classified as unknown for 48 cases that lacked audiologic data but had no other reported sequelae, we might have overestimated the proportion of survivors with sequelae. Our study revealed that 1 in 4 children did not receive an audiology assessment after meningococcal meningitis. Given this finding, we strongly recommend that children in New Zealand who are diagnosed with meningococcal meningitis receive audiology assessment before hospital discharge. Active follow-up for survivors of IMD should focus on confirming audiology assessment and screening for neurologic, developmental, and psychological effects (*2*,*9*). Our lack of access to mental health and educational data and shorter follow-up durations of ≥3 months might have underestimated the prevalence of long-term neurocognitive and psychological effects. Urgent action is needed to honor the nation’s commitment to Te Tiriti o Waitangi, the 1840 founding document that established bicultural partnership between indigenous Māori and the British Crown.

Our data demonstrate the usefulness of PCR for diagnosing culture-negative IMD (*32*,*33*). Blood culture results were negative in 79% of children who received prehospital antibiotics. However, when performed, *N. meningitidis* blood PCR was positive in all those cases. Drew et al. similarly reported positive blood PCR in 25 of 28 IMD cases that had a negative blood culture after intramuscular penicillin (*32*). We suggest that clinicians consider using *N. meningitidis* PCR testing, especially in the context of prior antibiotic administration. We found no statistically significant differences in clinical outcomes between children who received prehospital parenteral antibiotics and those who did not; however, our study was not powered to detect a difference in those outcomes. 

In our cohort, bacteremia and meningitis coexisted in 44.2% of cases; we propose that CSF testing be carefully considered for those with proven meningococcal bacteremia, especially in infants. In cases with bacteremia, 85.9% had a rash at first examination; rash characteristics included purpura (50.7%), petechiae without purpura (40.4%), and blanching only (8.9%), findings similar to those reported for a pediatric cohort in Ireland (*9*). Whereas a classic purpuric or petechial rash can suggest IMD, rash at presentation might be nonspecific or absent. It is therefore important for clinicians to maintain a high index of suspicion of IMD in children with suspected sepsis without rash.

In our cohort, we noted an increase over time in the proportion of isolates with reduced penicillin susceptibility. Similar trends have been reported among adults in Auckland, as well as in Spain and Australia (*34*–*36*). Earlier literature reported an association between reduced penicillin susceptibility and increased complications (*37*); however, no difference in outcomes were noted for our pediatric cohort or for the Auckland adult cohort (*34*). New Zealand guidelines recommend a third-generation cephalosporin for empiric treatment of sepsis in children (*38*). Because all isolates we studied were ceftriaxone-susceptible, reduced penicillin susceptibility is unlikely to have clinical significance for empiric therapy in New Zealand.

Our study illustrates the considerable inequity of IMD in the Auckland region of New Zealand. Māori and Pacific children had disproportionately higher rates of IMD and were more likely to experience complications. All but 1 death occurred in Māori or Pacific children. Children living in Auckland’s most deprived 20% of neighborhoods had rates of IMD 17 times higher than those in the least deprived 20% of neighborhoods. The relationship between ethnicity, socioeconomic deprivation, and the risk of severe childhood infections is not well understood but is likely rooted in the ongoing effects of colonization and structural racism (*39*). Recent findings from a nationally representative longitudinal study, Growing Up in New Zealand (*40*), indicate that disparities in infectious disease hospitalizations among infants of Māori or Pacific peoples can be only partly explained by socioeconomic deprivation factors. Nonetheless, household crowding has been shown to be strongly associated with epidemic IMD in New Zealand (*41*). 

Addressing the upstream determinants of health is important, but vaccination remains the best strategy to control IMD and is a key method for reducing inequity (*4*,*5*,*42*). Although New Zealand’s universal vaccination programs have not yet resulted in equitable uptake, prioritizing delivery and implementation might improve coverage and outcomes for those most at risk (*43*). Despite having the highest rate of MenB in the world and some prior success with MeNZB immunization, New Zealand has not yet included 4CMenB in the National Immunization Schedule nor funded vaccine for children at highest risk of disease. The real-world evidence for 4CMenB is clear and demonstrates that control of IMD in New Zealand is within reach (*15*).

In conclusion, IMD remains a severe, life-threat¬ening disease in young children in New Zealand; Māori and Pacific infants and those living in areas of socioeconomic deprivation are at greatest risk. The recent increase in incidence of MenB IMD highlights the urgent case for inclusion of 4CMenB in the National Immunization Schedule. Using *N. meningitidis* PCR to aid diagnosis of culture-negative, clinically suspected IMD, along with routine inpatient audiology assessment after cases of meningococcal meningitis, may improve clinical outcomes.

Appendix Additional information about pediatric invasive meningococcal disease in Auckland, New Zealand, 2004–2020.
