# Supplementary material for: Pediatric Invasive Meningococcal Disease, Auckland, New Zealand (Aotearoa), 2004–2020
Source: Emerg Infect Dis. 2023 Apr;29(4):686–95. doi: 10.3201/eid2904.221397 (PMC10045698; doi:10.3201/eid2904.221397)
Supplement: Appendix — Additional information about pediatric invasive meningococcal disease in Auckland, New Zealand, 2004–2020. [file 22-1397-Techapp-s1.pdf]

Article DOI: http://doi.org/10.3201/eid2904.221397

Pediatric Invasive Meningococcal Disease, Auckland, New Zealand (Aotearoa), 2004–2020

Appendix

Appendix Table 1. Comparison of *Neisseria meningitidis* culture and PCR in 319 confirmed cases of invasive meningococcal disease in children <15 years of age, Auckland, New Zealand, 2004–2020

Measure	Blood PCR in all IMD cases, n = 163		
	Positive	Negative	*Total*
N. meningitidis blood culture in all IMD cases, n = 318			
Positive	61	2	*63*
Negative	94	6	*100*
Total	*155*	*8*	*163*
	CSF PCR in all meningococcal meningitis cases, n = 84		
N. meningitidis CSF culture in meningococcal meningitis cases, n = 137			
Positive	13	0	*13*
Negative	65	5	*70*
Total	*78*	*5*	*83*

*CSF, cerebrospinal fluid; IMD, invasive meningococcal disease.

Appendix Table 2. Laboratory and neuroimaging characteristics in 319 confirmed cases of invasive meningococcal disease in children <15 years of age, Auckland, New Zealand, 2004–2020*

Parameter	No. cases with abnormal value for age (%)	Median (IQR)
Blood, n = 317		
Leukocyte count, n = 317†	186/317 (58.7)	15.5 x 10^9/L (8.9–22.4 x10^9/L)
Leukocytosis	152/317 (47.9)	NA
Leukopenia	34/317 (10.7)	NA
Platelets, n = 316‡	73/316 (23.1)	242 x 10^9/L (171–325 x10^9/L)
Thrombocytopenia	63/316 (19.9)	NA
Thrombocytosis	10/316 (3.2)	NA
Lactate, ref. range 0.5–2.2 mmol/L, n = 120	91/120 (75.8)	3.3 mmol/L (2.3–5.8 mmol/L)
Prothrombin ratio, ref. range 0.8–1.2, n = 181	109/181 (60.2)	1.3 (1.2–1.6)
C-reactive protein, n = 181§	171/181 (94.5)	104 mg/L (47–164 mg/L)
Creatinine, n = 301¶	127/301 (42.2)	44 µmol/L (35–60 µmol/L)
Bilirubin, n = 157#	9/157 (5.7)	9 µmol/L (6–15 µmol/L)
CSF, reference range; n = 138		
Leukocyte count**	130/133 (97)	4,660 x 10^6/L (340–6400 x10^6/L)
Polymorphs, 0%	123/126 (98)	85% (74%–90%)
Protein, 0.15–0.45 g/L	110/130 (85)	1.08 g/L (0.56–2.24 g/L)
Glucose, 2.8–4.4 mmol/L	68/129 (53)	NA
<0.5 mmol/L††	26/129 (20)	NA
≥0.5 mmol/L	42/129 (33)	3 mmol/L (2.0–3.8 mmol/L)
Positive Gram stain	57/133 (43)	NA
Neuroimaging		
Any imaging study	87/319 (27)	NA
Computed tomography	87/319 (27)	NA
Magnetic resonance imaging	27/319 (8.5)	NA
Abnormal neuroimaging results, n = 87	34/87 (39)	NA
Meningitis, cerebritis, ventriculitis	23/87 (26)	NA
Cerebral ischemia, infarction, hemorrhage	16/87 (18)	NA
Subdural empyema	11/87 (13)	NA
Hydrocephalus, raised intracranial pressure	9/87 (10)	NA

Parameter	No. cases with abnormal value for age (%)	Median (IQR)
Other abnormal neuroimaging‡‡	14/87 (16)	NA

*CSF, cerebrospinal fluid; IQR, interquartile range; NA, not applicable.
†Leukocyte count reference ranges: >34 x10^9/L age <1 wk; 5–19.5 x 10^9/L age 1wk–1 mo; 5–17.5 x 10^9/L age 1 mo–1 yr; 6–15.5 x 10^9/L age 2–5yr; 4.5–13.5 x 10^9/L age 6–12 yr; 4.5–11 x 10^9/L age 13 to <18 yr.
‡Platelet count reference ranges: 150–400 x 10^9/L age <1mo; 150–575 x 10^9/L age 1–11 mo; 150–500 x 10^9/L age 1–4 yr; 150–475 x 10^9/L age 5–8 yr, 150–425 x 10^9/L age 9–13 yr, 150–400 x 10^9/L age >13 yr.
§C-reactive protein reference ranges: <10 mg/L age <3 mo; <8 mg/L age 3 mo–15yr.
¶Creatinine reference ranges: <95 µmol/L age <1 wk; <50 µmol/L age 1–4 wk; <40 µmol/L age 4 wk–2 yr; <50 µmol/L age 3–6 yr; <65 µmol/L age 7–12 yr; <80 µmol/L age 13–15 yr.
#Bilirubin reference ranges: <150 µmol/L age <1 d; <200 µmol/L age 1 d, <250 µmol/L age 2 d; <300 µmol/L age 3–6 d; <100 µmol/L age 1–2 wk; <50 µmol/L age 3 wk; <25 µmol/L age ≥4 wk.
**CSF leukocyte count reference ranges: <20 x10^6/L age <1 mo; <5 x10^6/L age ≥1 mo.
††CSF glucose counts below 0.5 mmol/L reported as: <0.5 mmol/L.
‡‡Other abnormal neuroimaging results included cerebral venous sinus thrombosis or other vascular abnormality 4/87 (4.6%); septic emboli 4/87 (4.6%); cerebral atrophy 3/87 (3.4%); cochlear or other cranial nerve enhancement 2/87 (2.3%); and thalamic gliosis 1/87 (1.1%).

Appendix Table 3. Characteristics of 13 fatal cases of invasive meningococcal disease in children <15 years of age, Auckland, New Zealand, 2004–2020*

Year†	Group	Age, mo/sex	Ethnicity	NZDep quintile‡	MeNZB status, no. vaccines	Days from last MeNZB	MeNZB subtype IMD§	Bacteremia	Meningitis	Days from admission	Cause of death
2004	B	9/M	Pacific	5	None	NA	Y	Y	Y	29	Neurologic
2005	C	64/F	Māori	5	Complete,3	84	N	N	Y	1	Sepsis
2006	B	3/M	Pacific	5	Partial, 1	45	Y	Y	N	0	Sepsis
2007	B	3/F	Pacific	5	Partial, 1	8	Y	Y	N	0	Sepsis
2007	B	4/M	Asian	5	Partial, 2	24	Y	Y	N	2	Sepsis
2008	C	18/M	Māori	3	Complete, 4	213	N	Y	Y	0	Sepsis
2009	C	5/M	Māori	5	None	NA	N	Y	Y	13	Sepsis
2014	B	42/F	Pacific, European	5	None	NA	Y	Y	No	1	Sepsis
2017	B	4/M	Māori	5	None	NA	Y	Y	N	1	Sepsis
2018	B	2/F	Pacific	4	None	NA	N	Y	Y	0	Sepsis
2018	W	4/M	Pacific	5	None	NA	N	Y	Y	0	Sepsis
2018	W	103/M	Pacific	5	None	NA	N	Y	N	1	Sepsis
2019	B	3/M	Māori, Pacific	5	None	NA	N	Y	Y	10	Neurologic

*IMD, invasive meningococcal disease; NZDep, New Zealand Deprivation Index
†The MeNZB immunization program concluded in 2008.
‡Each NZDep quintile contains ≈20% of the population. Quintile 1 = least deprived; quintile 5 = most deprived.
§IMD subtypes against which MeNZB vaccination is expected to provide protection.

Appendix Table 4. Relationship between MeNZB vaccination status and timing of presentation of MeNZB subtype invasive meningococcal disease in 137 children <15 years of age, Auckland, New Zealand, 2004–2020

MeNZB vaccination status	No. cases (%)	Mean no. days between last vaccine and illness (95% CI)	p value
No. doses before illness			
0	82 (60.0)	NA	0.0003
1	11 (8.0)	37 (20–54)	
2	7 (5.1)	68 (41–178)	
3	29 (21.2)	568 (386–751)	
≥4	8 (5.8)	642 (278–1,005)	
MeNZB schedule			
Ineligible*	40 (29.2)	NA	<0.0001
Unvaccinated	42 (30.7)	NA	
Partially vaccinated	24 (17.5)	137 (3–271)	
Complete	31 (22.6)	620 (457–782)	
Mean difference complete vs. partial	NA	483 (277–689)	<0.0001

*Not eligible due to completion of MeNZB immunization program.
